# Morphological characteristics of seed starch granules of Fagaceae in South China and their implication in paleodiet

**DOI:** 10.3389/fpls.2022.977152

**Published:** 2022-11-14

**Authors:** Tong Yu, Qing Yang, Min Deng, Nan Cheng, Kaiping Yao, Wanshu Yang, Xueping Ji, Hongbo Zheng

**Affiliations:** ^1^ Yunnan Key Laboratory of Earth System Science, Yunnan University, Kunming, China; ^2^ Key Laboratory of Digital Human Technology R&D and Application of Yunnan Provincial Department of Education, School of History and Archival Science, Yunnan University, Kunming, China; ^3^ School of Ecology and Environmental Science, Yunnan University, Kunming, China; ^4^ Kunming Institute of Zoology, Chinese Academy of Sciences, Kunming Natural History Museum, Kunming, China; ^5^ School of Earth and Environmental Sciences, The University of Queensland, Bribane, QLD, Australia

**Keywords:** South China, nut fruits, *Quercus*, morphological characteristics, starch granule

## Abstract

Nut fruits likely played a significant role before and during the origin of agriculture; however, relatively little research conducted on the morphological characteristics and statistical comparisons of nut fruit starch granule hinders the progress of paleodietary analysis of prehistorical society. For better species identification of starch granule remaining on tools discovered at archaeological sites, it is desirable to develop a more abundant morphology database of modern nut fruit starch granules as well as the establishment of relevant identification standards. Therefore, nuts from 40 species in four genera (*Quercus*, *Lithocarpus*, *Castanea*, and *Castanopsis*) of Fagaceae were collected from South China for statistical measurement and comparative analysis. Starch granules are highly accumulated in 34 species except for 6 species, whose shapes involve oval, subcircular, drop-shaped, rounded triangle, polygonal, spherical caps, and bell-shaped types, or a combination of several types, and the average length is between 10 and 20 μm. According to research on *Quercus* phylogeny relationships, it was found that the species in the same infragenious section produce similar morphological characteristics of starch granules. The result was applied in the identification of starch granules extracted from stone tools from the 20 to 10 ka cultural layer of Xiaodong Rockshelter, and some starch granules can be recognized to species level, revealing that nuts from *Quercus* and *Lithocarpus* were gathered and exploited by ancient people. This expansion of modern starch presentation and comparison of nuts helps to improve the accuracy of the identification of ancient starch and deepen the understanding of plant utilization of ancient humans.

## 1 Introduction

The utilization of plants and animals is the foundation of livelihood and development of ancient human society, learning which can assist us in comprehending the history of human civilization. Domestication and the origin of agriculture furnished a physical foundation in demographic development and dispersal (Liu and Chen, 2012). Currently, the prevailing opinion is that North China is the original region of rain-fed agriculture, with foxtail millet (*Setaria italica*) and broomcorn millet (*Panicum miliaceum*) as major crops (Lu et al., 2009; Zhang et al., 2011a; Liu et al., 2011; Yang et al., 2012a; Yang et al., 2012b; Zhao, 2014; Yang et al., 2015; Liu et al., 2015), and the Yangtze River valley is another center of agricultural origin, represented by the cultivation of rice (Bai and Su, 1994; Zhao, 1998; Liu et al., 2007; Jones and Liu, 2009; Huan et al., 2014; Wu et al., 2014; Tao, 2016; Zhao, 2019; Zhao, 2020). Combined with international archaeological and anthropological research data (Anderson, 1980; Mason, 1992; Mason, 1996; Bettinger et al., 1997; Haslam, 2004; Bellwood, 2005), Liu has speculated that acorns may additionally have been a major source of starchy food in many areas prior to the development of China’s prehistory cereal agriculture in the Late and Middle Neolithic (Liu, 2008; Liu, 2010). The main function of Chinese grinding stone tools was to process nuts such as acorns rather than grains (Zhao, 2006; Cui, 2018), and the gathering, processing, and consumption of nuts (especially acorns) contributed to the settlement of human society (Sun et al., 1981; Wang, 2006; Jiang, 2007; Yang and Jiang, 2010), while the manufacturing of cultivated rice and foxtail millet took a secondary position for a long time.

Analyzing the microfossils remaining on the giant quantity of stone tools and pottery excavated from the site permits us to study ancient plant utilization. The plant microfossils primarily include pollen, phytolith, and starch granule. Pollen is solely recognized to the genera level, with an ambiguous indication of human utilization on plant sources; phytoliths are effective in determining particular species such as Poaceae; however, their indicator value for human utilization on woody plants is unclear (Wang and Lü, 1993; Hodson et al., 2005; Ge et al., 2020). In contrast, the richness of starch granule in plant fruits and root tubers, their long-term maintenance in archaeological remains and strata, and the variation in morphological characteristics of different species and genera provide potentially clear indications of human consumption of certain plant resources (Burrell, 2003).

The starch granule morphology varies among different genera (Yang et al., 2006). Starch is a long-chain compound formed by glucose molecules and is stored in organs such as roots, stems, and seeds of plants in the form of starch granule. The formation of starch granule starts at a point called the hilum with additional layers laid down (Tester, 1997). The size of starch granule generally varies from approximately 1 micron to 100 microns (Sivak and Preiss, 1998), and an extinction cross can be viewed with polarized light (Sterling, 1984; Dziezak, 1988; Yiu, 1993). Starch granules are classified as simple, compound, or semi-compound depending on how they are formed in the amyloplast (Banks and Greenwood, 1975). Therefore, according to the morphological characteristics of starch granules, they can be classified to determine plant genera and species.

The method of starch granule analysis has been widely applied to studies on the origin and spread of crops, human utilization on plants, and the functional analysis of stone tools in the Americas, Australia, and East and West Asia (Fullagar, 1998; Piperno and Holst, 1998; Piperno et al., 2000; Pearsall et al., 2004; Piperno et al., 2004; Torrence and Barton, 2006; Perry et al., 2007; Li et al., 2010; Zhang et al., 2011b; Hart, 2014; Lü et al., 2014; Ma et al., 2014; Hardy et al., 2016; García-Granero et al., 2017; Wang et al., 2017; Wang et al., 2019). Starch granule analysis of the grooved basin from the Lingjiatan site, Hanshan County, Anhui Province (Yang et al., 2009b), and of grinding stone tools at the Shangzhai site, Beijing (Sun et al., 2019), revealed that the ancient humans had consciously exploited acorns.

Acorns refer to nuts of the Fagaceae family that can be exploited except for chestnut, which is widely cultivated. Fagaceae plants are mainly found in the tropics and subtropics of the Northern Hemisphere (Hu et al., 2000). The acorn is highly adaptable, with a broad distribution in China, where the annual production is estimated at 6–7 billion kg (Xie and Xie, 2002). Meanwhile, acorns are rich in nutrients, including starch, tannin, protein, oil, amino acids, and minerals (Ao et al., 1998). Acorns are suitable for eating after desiccation and can be used in brewing (Hou and Wang, 1996), textiles, and so on (Du and Li, 1996; He et al., 2003).

However, studies of the starch granule morphology of modern nuts lack detailed and clear identification for archaeological research (Xu and Gao, 2004). Chinese scholars have identified starch granule from several genera of Fagaceae in northern China (Yang et al., 2009a), but the starch statistics of edible species from South China, where plant resources are rich, are still desired, which significantly hinders the study of ancient plant utilization in the region. In this paper, nuts of 40 species of Fagaceae from the southern part of China were collected for the presentation and comparison of starch granule morphological characteristics and statistical analysis, providing a reliable identification key for starch granule extracted from archaeological sites and thus revealing the use of plant resources by ancient people in South China.

## 2 Materials and methods

### 2.1 Modern starch reference collections

**Figure 1:**
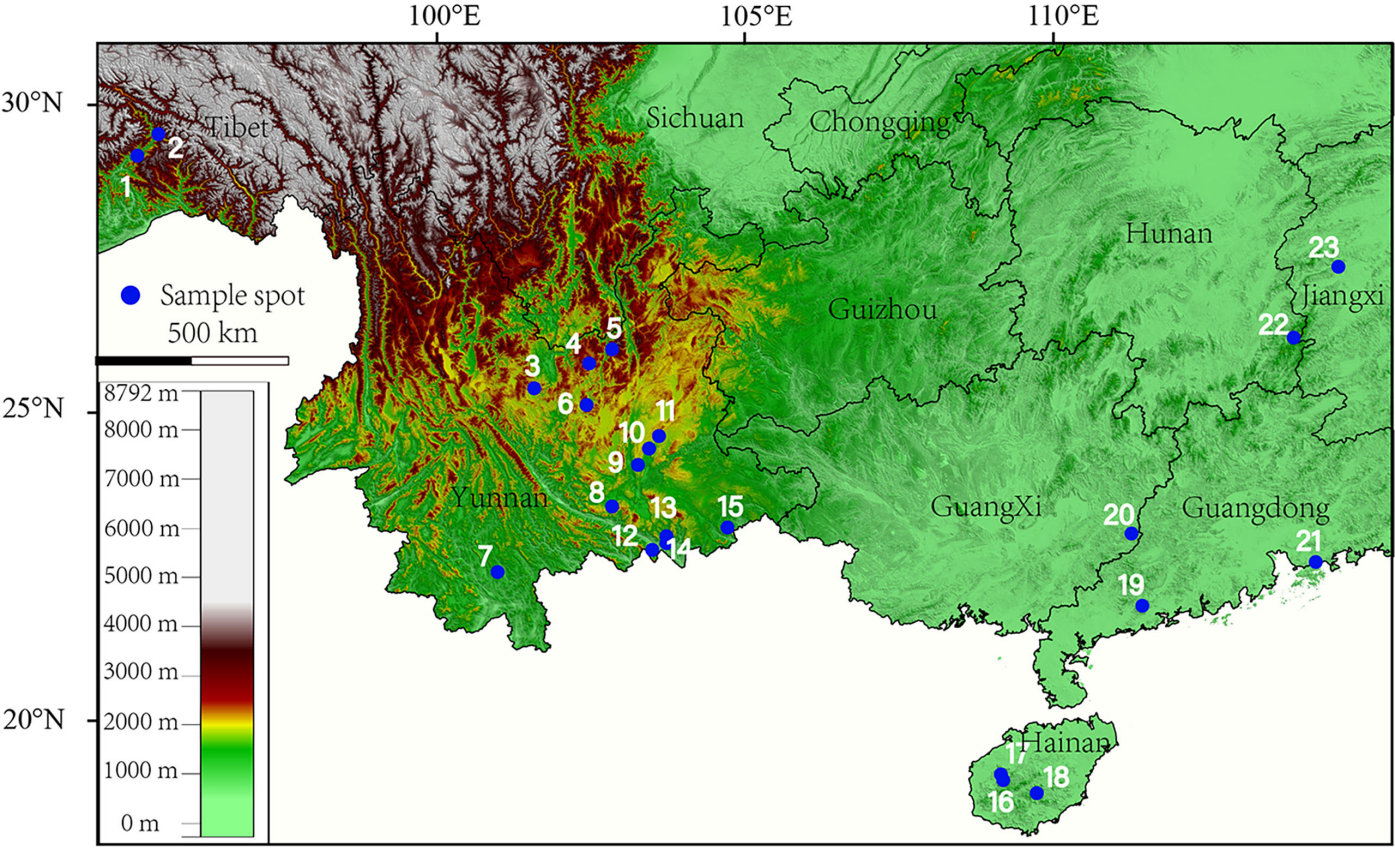
Sampling spots of specimens used in this study (1: *Quercus kiukiangensis*; 2: *Quercus lamellosa*; 3: *Lithocarpus dealbatus*; 4: *Quercus schottkyana, Quercus variabilis, Quercus serrata, Quercus aliena, Lithocarpus elizabethiae, Castanea seguinii, Castanopsis orthacantha*; 5: *Lithocarpus craibianus*; 6: *Quercus longispica*; 7: *Quercus rex*; 8: *Lithocarpus mairei*; 9: *Quercus franchetii*; 10: *Quercus cocciferoides*; 11: *Quercus argyrotricha*; 12: *Lithocarpus gymnocarpus*; 13: *Lithocarpus pachylepis*; 14: *Quercus augustinii, Lithocarpus xylocarpus*; 15: *Quercus marlipoensis, Lithocarpus balansae, Lithocarpus c.f. annamitorus, Lithocarpus bacgiangensis, Lithocarpus longinux, Lithocarpus sp.1*; 16: *Quercus patelliformis, Lithocarpus longipedicellatus*; 17: *Quercus fleuryi, Lithocarpus fenzelianus*; 18: *Lithocarpus sp.2*; 19: *Lithocarpus longanoides*; 20: *Quercus blakei, Quercus phanera, Quercus kouangsiensis*; 21: *Quercus litseoides, Quercus sessilifolia*; 22: *Lithocarpus skanianus*; 23: *Quercus gilva*).

Forty species specimens of *Quercus, Lithocarpus, Castanea*, and *Castanopsis* were primarily collected during the fruit maturation period in September or October in 2020–2021, ranging from Yunnan, Hainan, Guangdong, Guangxi, Jiangxi Province, and Tibet Autonomous Regions, China (**Figure 1**), involving evergreen broad-leaved forest, tropical rainforest, hillside woodland, and woodland. The detailed collection information is summarized in **Supplementary Table S1**.

### 2.2 Method of extraction of starches from specimens

Starch granules of acorns were released by the following method: (1) the nut, in a small plastic sealed bag, was broken with a hammer; (2) small pieces of one broken nut were transported to new centrifugal tubes with pure water for 24 h of soaking; (3) after soaking, the samples were crushed with clean glass stirring rods to fully release the starches. Thirty microliters of the suspension was pipetted onto a clean slide and mounted in a 20%/80% glycerin/water solution. The prepared slides were observed under polarized and bright fields using a Leica DM4P microscope, and images were taken using the same system.

For statistical purposes, at least 100 granules of each specimen were recorded. Between 10 and 14 photographs were necessary to produce an adequate sample size when all granules on each photo were measured and characterized. We measured the longest orientable measurement through the hilum of each granule and recorded the following morphological features: granule shape; hilum position (eccentric/centric); the form of the fissure; presence or absence of lamellae; the form of the polarizing cross (cross-shaped/X-shaped) and surface texture (smooth/rough).

### 2.3 Electron microscopy scanning

The sample was dried at low temperature, tapped with a hammer to make a natural fracture, and attached to the sample desk with the section side down. Specimens were then gold coated, observed, and photographed by scanning electron microscopy (SEM) (Thermo Scientific Quattro S).

### 2.4 Image processing and data analysis

Photoshop 2021 software was used to choose individual granules, and their areas were determined. Then, the preprepared images were used for analysis in Image-Pro Plus as follows: granules were colored, and the image was made binary (starch white against black background). Then, each granule was counted, and the size was measured. Statistical analyses and plotting were performed using Origin Pro 2021.

### 2.5 Method of extraction and identification of starch granules from stone tools

Starch granules of stone tools were extracted by the following method: (1) brushing the stone tool to decontaminate; (2) using an ultrasonic machine with distilled water to remove the residues on the tool surface, the liquid was collected in a centrifuge tube; (3) adding 10 ml of deflocculant and shaking residue samples for 2 h to fully release the starches; and (4) residue samples were processed for starch extraction using the heavy liquid sodium polytungstate in a specific gravity of 1.8. Slides preparation and observation were the same with modern samples.

The morphological characteristics were observed and recorded, and the size of the ancient starch granule was measured. One-to-one comparisons with modern samples from the database were performed to identify the ancient starch grains. Reliable identification of archaeological starch grains is based on size, overall shape, position and form of the hilum, fissure, the presence or absence of lamellae, and the appearance and projection of the Maltese cross under a polarized microscope (Torrence et al., 2004; Wilson et al., 2010; Reichert, 1913).

## 3 Results

**Figure 2:**
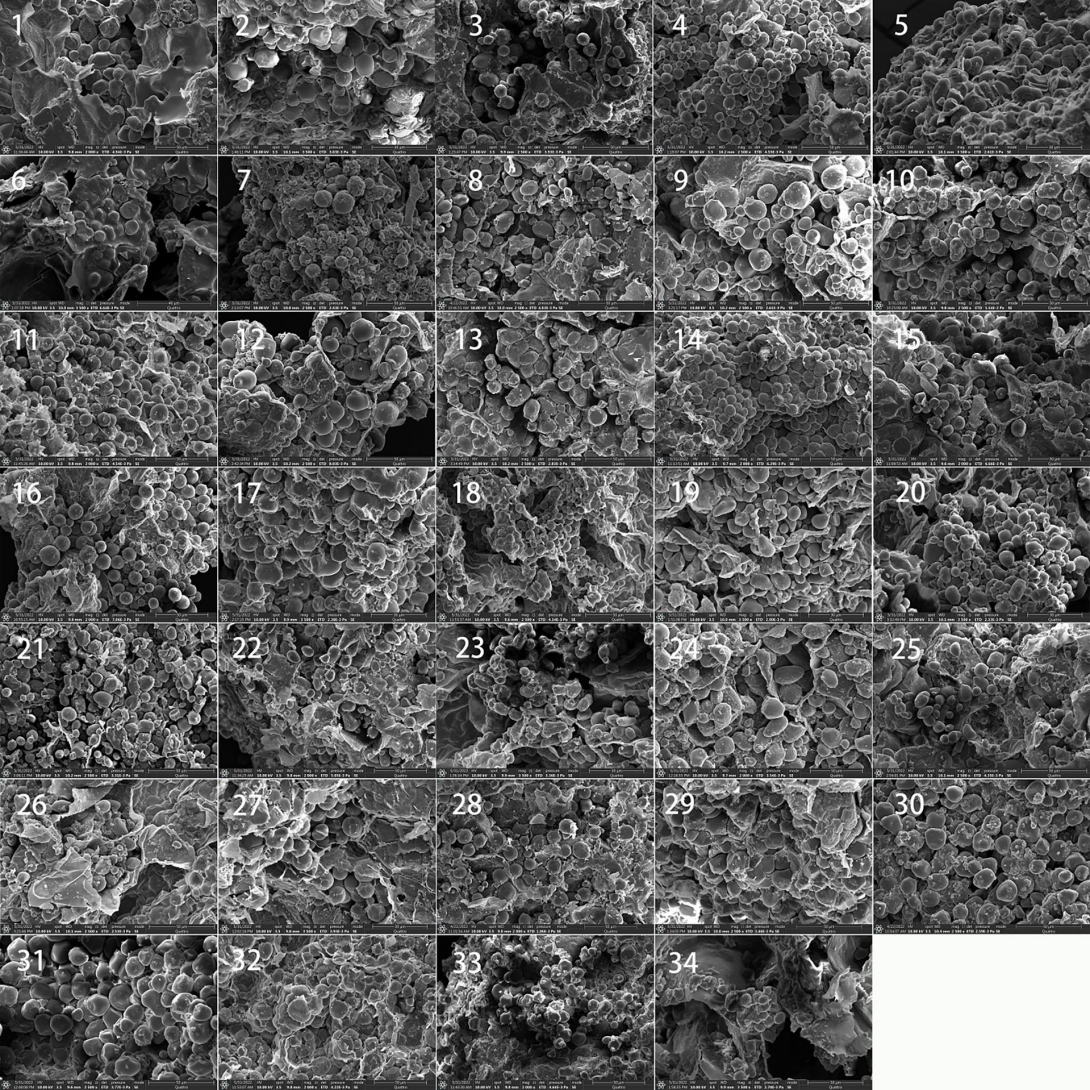
Scanning electron micrographs of starch granule in nuts of 34 species from Fagaceae. 1, *Quercus blakei;* 2, *Quercus argyrotricha*; 3, *Quercus augustinii*; 4, *Quercus litseoides*; 5, *Quercus kouangsiensis*; 6, *Quercus fleuryi*; 7, *Quercus lamellosa*; 8, *Quercus serrata*; 9, *Quercus sessilifolia*; 10, *Quercus gilva*; 11, *Lithocarpus elizabethiae*; 12, *Quercus kiukiangensis*; 13, *Quercus schottkyana*; 14, *Lithocarpus pachylepis*; 15, *Lithocarpus longipedicellatus*; 16, *Lithocarpus* sp.; 17, *Lithocarpus xylocarpus*; 18, *Lithocarpus fenzelianus*; 19, *Quercus longispica*; 20, *Quercus marlipoensis*; 21, *Castanea seguinii*; 22, *Quercus cocciferoides*; 23, *Quercus franchetii*; 24, *Quercus phanera*; 25, *Quercus aliena*; 26, *Quercus variabilis*; 27, *Lithocarpus craibianus*; 28, *Lithocarpus longanoides*; 29, *Lithocarpus mairei*; 30, *Lithocarpus balansae*; 31, *Castanopsis orthacantha*; 32, *Lithocarpus skanianus*; 33, *Lithocarpus dealbatus*; 34, *Lithocarpus longinux*.

All 40 samples were observed, and each produced slides with over 100 granules. A detailed description of the morphological characteristics of the starch granule for each species is given in **Table 1**. We note that for six species, no identifiable starch granules were observed. However, according to the research, *Quercus rex, Quercus patelliformis*, and *Lithocarpus gymnocarpus* are rich in starch, particularly *L. gymnocarpus*, which has been found to have 66.12% starch in the kernel. It is not yet clear why there is no starch in these samples in our study. Further work is required to clarify this issue. Scanning electron micrographs of starch granule in nuts of 34 species are displayed in **Figure 2**.

**Table 1:** Summary of starch granule dimensions and morphological features in 34 species from Fagaceae.

Infragenious groups/sections	Sample	Granule count	Shape	Length range (μm)	Mean length (μm)	Hilum	Fissures	Lamellae	Polarizing cross	Compound granules
Cyclobalanopsis	Quercus blakei	169	Oval, bell-shaped	7.84–30.10	17.11 ± 4.43	Eccentric	None	None	X-shaped	None
Cyclobalanopsis	Quercus kouangsiensis	157	Oval, subcircular	5.86–18.37	11.38 ± 2.21	Eccentric or centric	None	None	X-shaped or cross-shaped	12.7%
Cyclobalanopsis	Quercus argyrotricha	121	Oval, bell-shaped	9.41–22.50	14.67 ± 3.02	Eccentric	None	None	X-shaped	3.3%
Cyclobalanopsis	Quercus gilva	331	Subcircular, bell-shaped	9.12–19.67	12.67 ± 1.80	Eccentric or centric	Linear-shaped	None	X-shaped or cross-shaped	37%
Cyclobalanopsis	Quercus kiukiangensis	178	Subcircular, oval, bell-shaped	8.98–28.10	16.3 ± 4.67	Eccentric	Linear-shaped	None	X-shaped or cross-shaped	None
Cyclobalanopsis	Quercus phanera	101	Drop-shaped	6.17–33.67	17.35 ± 5.96	Eccentric	None	None	X-shaped	None
Cyclobalanopsis	Quercus schottkyana	152	Subcircular, oval, bell-shaped	6.28–20.73	12.93 ± 3.87	Eccentric or centric	None	None	X-shaped or cross-shaped	None
Cyclobalanopsis	Quercus sessilifolia	148	Oval	7.67–26.63	17.06 ± 4.46	Eccentric	None	None	X-shaped	None
Cyclobalanopsis	Quercus augustinii	132	Oval, bell-shaped	7.87–24.91	14.02 ± 3.52	Eccentric	None	None	X-shaped	None
Cyclobalanopsis	Quercus litseoides	132	Oval, bell-shaped	7.46–16.89	11.83 ± 2.32	Eccentric	None	None	X-shaped	3.8%
Cyclobalanopsis	Quercus lamellosa	108	Subcircular, oval, polygonal	3.53–20.24	11.25 ± 3.72	Eccentric	None	None	X-shaped	2%
Cyclobalanopsis	Quercus fleuryi	101	Subcircular, oval	4.57–13.37	8.21 ± 1.75	Centric	None	None	X-shaped or cross-shaped	None
Quercus	Quercus cocciferoides	152	Drop-shaped, subcircular	5.93–30.06	18.68 ± 5.29	Eccentric or centric	Linear-shaped	None	Cross-shaped	None
Quercus	Quercus serrata	151	Oval, rounded triangle	5.99–19.44	11.31 ± 2.65	Eccentric	Linear-shaped	None	X-shaped	None
Quercus	Quercus aliena	175	Oval, rounded triangle	6.81–16.83	11.24 ± 2.34	Eccentric	Linear-shaped	None	Faint	None
Quercus	Quercus variabilis	108	Drop-shaped, rounded triangle	7.59–27.44	12.72 ± 4.12	Eccentric	Linear-shaped or Y-shaped	None	Faint	None
Quercus	Quercus franchetii	110	Drop-shaped	7.01–25.76	13.44 ± 4.00	Eccentric	None	None	Faint	None
Quercus	Quercus longispica	123	Drop-shaped, oval, polygonal, bell-shaped, subcircular	6.08–33.97	15.92 ± 5.76	Eccentric	Linear-shaped, V-shaped, or X-shaped	None	X-shaped	2%
Quercus	Quercus marlipoensis	172	Drop-shaped, oval, polygonal, subcircular	5.11–28.21	12.55 ± 4.66	Eccentric	None	None	Cross-shaped or X-shaped	None
Lithocarpus	Lithocarpus dealbatus	179	Spherical caps, oval, polygonal, circular	8.01–31.75	17.23 ± 4.58	Eccentric	None	None	Cross-shaped	10.6%
Lithocarpus	Lithocarpus craibianus	145	Polygonal, subcircular	10.27–27.81	17.47 ± 3.72	Centric	None	None	Cross-shaped	None
Lithocarpus	Lithocarpus mairei	104	Polygonal, bell-shaped, subcircular	9.59–27.01	18.30 ± 4.20	Centric	Linear-shaped or X-shaped	None	Cross-shaped or X-shaped	2%
Lithocarpus	Lithocarpus pachylepis	140	Subcircular, polygonal, bell-shaped	5.54–16.44	10.28 ± 2.32	Centric	Linear-shaped or X-shaped	None	Cross-shaped or X-shaped	9%
Lithocarpus	Lithocarpus sp.	105	Subcircular, polygonal, bell-shaped	3.21–24.25	11.54 ± 4.93	Centric	None	None	Cross-shaped or X-shaped	1%
Lithocarpus	Lithocarpus elizabethiae	230	Subcircular, bell-shaped	5.15–21.43	10.10 ± 2.67	Centric	Linear-shaped or X-shaped	None	Cross-shaped	1%
Lithocarpus	Lithocarpus longipedicellatus	105	Subcircular, polygonal, bell-shaped	4.31–30.63	14.40 ± 5.48	Centric	None	None	Cross-shaped or X-shaped	2%
Lithocarpus	Lithocarpus fenzelianus	124	Subcircular	4.62–12.98	8.35 ± 1.94	Centric	None	None	Cross-shaped	None
Lithocarpus	Lithocarpus balansae	131	Polygonal, oval, subcircular	10.71–30.46	19.04 ± 4.10	Eccentric or centric	None	Well-defined	X-shaped	6.9%
Lithocarpus	Lithocarpus skanianus	165	Polygonal, bell-shaped, subcircular	10.50–41.47	19.35 ± 5.04	Eccentric or centric	None	Well-defined	Cross-shaped or X-shaped	2.4%
Lithocarpus	Lithocarpus longanoides	126	Polygonal, subcircular	9.32–24.07	15.41 ± 3.38	Centric	Linear-shaped	Well-defined	Cross-shaped	4%
Lithocarpus	Lithocarpus xylocarpus	143	Oval, subcircular	7.21–22.47	13.23 ± 3.48	Eccentric or centric	None	None	Cross-shaped or X-shaped	None
Lithocarpus	Lithocarpus longinux	167	Bell-shaped, subcircular	5.57–10.47	7.70 ± 0.95	Centric	None	None	X-shaped	22.8%
Castanopsis	Castanopsis orthacantha	109	Polygonal, subcircular, bell-shaped, oval	8.69–22.60	16.16 ± 3.27	Centric	None	None	Cross-shaped	2%
Castanea	Castanea seguinii	150	Drop-shaped, subcircular, oval, polygonal	5.58–21.41	13.31 ± 3.93	Eccentric or centric	None	None	X-shaped	3%
	Quercus rex	—	—	—	—	—	—	—	—	—
Lithocarpus	Lithocarpus sp.	—	—	—	—	—	—	—	—	—
Quercus	Quercus patelliformis	—	—	—	—	—	—	—	—	—
Lithocarpus	Lithocarpus gymnocarpus	—	—	—	—	—	—	—	—	—
Lithocarpus	Lithocarpus bacgiangensis	—	—	—	—	—	—	—	—	—
Lithocarpus	Lithocarpus c.f. annamitorus	—	—	—	—	—	—	—	—	—

### 3.1 Starch granules from *Quercus*

The detailed description of starch morphological characteristics from 19 species of *Quercus* are as follows.

#### 3.1.1 Starch granules from *Quercus blakei*

The starch granules from *Quercus blakei* in our study are divided into two groups according to shape. One is the oval type, which accounts for 61%, with a smooth surface and invisible lamellae, whose mean size is 19.90 ± 3.18 μm. The other is the bell-shaped type, which accounts for 39%, with a smooth surface and invisible lamellae, whose mean size is 12.88 ± 2.25 μm.

#### 3.1.2 Starch granules from *Quercus kouangsiensis*

The starch granules from *Quercus kouangsiensis* in our study are divided into two groups according to shape. One is the oval type, which accounts for 78.3%, with some small hollows on the surface and invisible lamellae, whose mean size is 11.48 ± 2.19 μm. The other is the subcircular type, which accounts for 9%, with a smooth surface and invisible lamellae, whose mean size is 10.51 ± 2.19 μm. Meanwhile, compound granule accounts for 12.7%.

#### 3.1.3 Starch granules from *Quercus argyrotricha*

The starch granules from *Quercus argyrotricha* in our study are divided into two groups according to shape. One is the oval type, which accounts for 53.7%, with some small hollows on the surface and invisible lamellae, whose mean size is 15.99 ± 3.04 μm. The other is the bell-shaped type, which accounts for 43%, with a smooth surface and invisible lamellae, whose mean size is 13.62 ± 2.58 μm. Meanwhile, compound granule accounts for 3.3%.

#### 3.1.4 Starch granules from *Quercus gilva*

The starch granules from *Quercus gilva* in our study are divided into two groups according to shape. One is the subcircular type, which accounts for 31%, with a smooth surface, linear-shaped fissures through the hila, and invisible lamellae, whose mean size is 12.59 ± 1.79 μm. The other is the bell-shaped type, which accounts for 32%, with a smooth surface and invisible lamellae, whose mean size is 12.76 ± 1.80 μm. Meanwhile, compound granule accounts for 37%.

#### 3.1.5 Starch granules from *Quercus kiukiangensis*

The starch granules from *Quercus kiukiangensis* in our study are divided into three groups according to shape. The first group is the subcircular type, which accounts for 34.9%, with a smooth surface, linear-shaped fissures through the hila, and invisible lamellae, whose mean size is 13.23 ± 2.71 μm. The second group is the bell-shaped type, which accounts for 37.6%, with a smooth surface and invisible lamellae, whose mean size is 14.92 ± 3.12 μm. The last group is the oval type, which accounts for 27.5%, with a smooth surface, concave toward the distal end, and invisible lamellae, whose mean size is 22.06 ± 2.98 μm.

#### 3.1.6 Starch granules from *Quercus phanera*

The starch granules from *Quercus phanera* in our study is the drop-shaped type with a smooth surface and invisible lamellae, whose mean size is 17.35 ± 5.96 μm.

#### 3.1.7 Starch granules from *Quercus schottkyana*

The starch granules from *Quercus schottkyana* in our study are divided into three groups according to shape. The first group is the subcircular type, which accounts for 32.9%, with a smooth surface and invisible lamellae, whose mean size is 10.27 ± 2.14 μm. The second group is the bell-shaped type, which accounts for 29.6%, with a smooth surface and invisible lamellae, whose mean size is 10.80 ± 2.56 μm. The last group is the oval type, which accounts for 37.5%, with some small hollows on the surface, concave toward the distal end, and invisible lamellae, whose mean size is 16.94 ± 2.21 μm.

#### 3.1.8 Starch granules from *Quercus sessilifolia*

The starch granules from *Quercus sessilifolia* in our study is the oval type with some small hollows on the surface and invisible lamellae, whose mean size is 17.06 ± 4.46 μm.

#### 3.1.9 Starch granules from *Quercus augustinii*

The starch granules from *Quercus augustinii* in our study are divided into two groups according to shape. One is the oval type, which accounts for 84.1%, with some small hollows on the surface and invisible lamellae, whose mean size is 14.37 ± 3.66 μm. The other is the bell-shaped type, which accounts for 15.9%, with smooth surface and invisible lamellae, whose mean size is 12.16 ± 1.83 μm.

#### 3.1.10 Starch granules from *Quercus litseoides*

The starch granules from *Quercus litseoides* in our study are divided into two groups according to shape. One is the oval type, which accounts for 80.3%, with some small hollows on the surface and invisible lamellae, whose mean size is 11.85 ± 2.43 μm. The other is the bell-shaped type, which accounts for 15.9%, with a smooth surface and invisible lamellae, whose mean size is 11.69 ± 1.69 μm. Meanwhile, compound granule accounts for 3.8%.

#### 3.1.11 Starch granules from *Quercus lamellosa*

The starch granules from *Quercus lamellosa* in our study are divided into three groups according to shape. The first group is the subcircular type, which accounts for 47%, with a smooth surface and invisible lamellae, whose mean size is 10.07 ± 3.24 μm. The second group is the polygonal type, which accounts for 5%, with a rough surface and invisible lamellae, whose mean size is 14.83 ± 3.81 μm. The last group is the oval type, which accounts for 48%, with a smooth surface and invisible lamellae, and with very few granules bearing X-shaped fissures through the hila, whose mean size is 12.01 ± 3.78 μm. Meanwhile, compound granule accounts for 2%.

#### 3.1.12 Starch granules from *Quercus fleuryi*

The starch granules from *Quercus fleuryi* in our study are divided into two groups according to shape. One is the oval type, which accounts for 59%, with a smooth surface and invisible lamellae, whose mean size is 8.79 ± 1.80 μm. The other is the subcircular type, which accounts for 41%, with a smooth surface and invisible lamellae, whose mean size is 7.36 ± 1.27 μm.

#### 3.1.13 Starch granules from *Quercus cocciferoides*

The starch granules from *Quercus cocciferoides* in our study are divided into two groups according to shape. One is the drop-shaped type, which accounts for 78%, with a smooth surface, linear-shaped fissures through the hila, and invisible lamellae, whose mean size is 20.83 ± 3.25 μm. The other is the subcircular type, which accounts for 22%, with a smooth surface and invisible lamellae, whose mean size is 10.89 ± 3.64 μm.

#### 3.1.14 Starch granules from *Quercus serrata*

The starch granules from *Quercus serrata* in our study are divided into two groups according to shape. One is the oval type, which accounts for 57.6%, with a smooth surface, protruding toward the distal end, and invisible lamellae, whose mean size is 12.37 ± 2.31 μm. The other is the rounded triangle type, which accounts for 42.4%, with a smooth surface, linear-shaped fissures through the hila, and invisible lamellae, whose mean size is 9.85 ± 2.39 μm.

#### 3.1.15 Starch granules from *Quercus aliena*

The starch granules from *Quercus aliena* in our study are divided into two groups according to shape. One is the oval type, which accounts for 15%, with some small hollows on the surface and invisible lamellae, whose mean size is 10.99 ± 2.33 μm. The other is the rounded triangle type, which accounts for 85%, with a smooth surface, linear-shaped fissures through the hila, and invisible lamellae, whose mean size is 10.8 ± 2.86 μm.

#### 3.1.16 Starch granules from *Quercus variabilis*

The starch granules from *Quercus variabilis* in our study are divided into two groups according to shape. One is the rounded triangle type, which accounts for 64.8%, with a smooth surface, linear-shaped or Y-shaped fissures through the hila, and invisible lamellae, whose mean size is 12.59 ± 4.12 μm. The other is the drop-shaped type, which accounts for 35.2%, with a smooth surface, linear-shaped fissures through the hila, and invisible lamellae, whose mean size is 12.95 ± 4.18 μm.

#### 3.1.17 Starch granules from *Quercus franchetii*

The starch granules from *Quercus franchetii* in our study is the drop-shaped type with a smooth surface and invisible lamellae, whose mean size is 13.44 ± 4.00 μm.

#### 3.1.18 Starch granules from *Quercus longispica*

The starch granules from *Quercus longispica* in our study are divided into five groups according to shape. The first group is the subcircular type, which accounts for 13%, with a smooth surface and invisible lamellae, whose mean size is 10.95 ± 2.93 μm. The second group is the polygonal type, which accounts for 15%, with a rough surface and invisible lamellae, whose mean size is 21.74 ± 4.74 μm. The third group is the oval type, which accounts for 23%, with a smooth surface and invisible lamellae, and with very few granules bearing X-shaped or linear-shaped fissures through the hila, whose mean size is 14.38 ± 3.16 μm. The fourth group is the drop-shaped type, which accounts for 35%, with a smooth surface and invisible lamellae, and with very few granules bearing V-shaped fissures through the hila, whose mean size is 18.13 ± 5.68 μm. The last group is the bell-shaped type, which accounts for 13%, with a smooth surface and invisible lamellae, whose mean size is 11.12 ± 3.57 μm. Meanwhile, compound granule accounts for 2%.

#### 3.1.19 Starch granules from *Quercus marlipoensis*

The starch granules from *Quercus marlipoensis* in our study are divided into four groups according to shape. The first group is the subcircular type, which accounts for 17%, with a smooth surface and invisible lamellae, whose mean size is 7.55 ± 1.52 μm. The second group is the polygonal type, which accounts for 6%, with a rough surface and invisible lamellae, whose mean size is 11.89 ± 4.19 μm. The third group is the oval type, which accounts for 32%, with a smooth surface and invisible lamellae, whose mean size is 11.51 ± 4.12 μm. The last group is the drop-shaped type, which accounts for 45%, with a smooth surface and invisible lamellae, whose mean size is 15.32 ± 3.93 μm.

### 3.2 Starch granules from *Lithocarpus*

The detailed description of starch morphological characteristics from 13 species of *Lithocarpus* are as follows.

#### 3.2.1 Starch granules from *Lithocarpus dealbatus*

The starch granules from *Lithocarpus dealbatus* in our study are divided into four groups according to shape. The first group is the circular type, which accounts for 24%, with a smooth surface, concave hila, and invisible lamellae, whose mean size is 15.52 ± 3.39 μm. The second group is the polygonal type, which accounts for 21.8%, with a rough surface and invisible lamellae, whose mean size is 20.19 ± 3.55 μm. The third group is the oval type, which accounts for 20.7%, with a smooth surface, concave hila, and invisible lamellae, whose mean size is 19.40 ± 3.79 μm. The last group is the spherical caps type, which accounts for 22.9%, with a smooth surface and invisible lamellae, whose mean size is 14.37 ± 4.73 μm. Meanwhile, compound granule accounts for 10.6%.

#### 3.2.2 Starch granules from *Lithocarpus craibianus*

The starch granules from *Lithocarpus craibianus* in our study are divided into two groups according to shape. One is the polygonal type, which accounts for 59.3%, with a rough surface and invisible lamellae, whose mean size is 18.99 ± 3.27 μm. The other is the subcircular type, which accounts for 40.7%, with a smooth surface and invisible lamellae, whose mean size is 15.25 ± 3.19 μm.

#### 3.2.3 Starch granules from *Lithocarpus mairei*

The starch granules from *Lithocarpus mairei* in our study are divided into three groups according to shape. The first group is the subcircular type, which accounts for 7%, with a smooth surface, concave hila, and invisible lamellae, whose mean size is 18.36 ± 4.17 μm. The second group is the polygonal type, which accounts for 82%, with a rough surface, X-shaped or linear-shaped fissures through the hila, and invisible lamellae, whose mean size is 18.53 ± 4.33 μm. The last group is the bell-shaped type, which accounts for 11%, with a smooth surface, concave hila, and invisible lamellae, whose mean size is 16.64 ± 2.98 μm. Meanwhile, compound granule accounts for 2%.

#### 3.2.4 Starch granules from *Lithocarpus pachylepis*

The starch granules from *Lithocarpus pachylepis* in our study are divided into three groups according to shape. The first group is the subcircular type, which accounts for 68%, with a smooth surface, X-shaped or linear-shaped fissures through the hila, and invisible lamellae, whose mean size is 9.97 ± 2.11 μm. The second group is the polygonal type, which accounts for 19%, with a rough surface, X-shaped fissures through the hila, and invisible lamellae, whose mean size is 11.39 ± 2.57 μm. The last group is the bell-shaped type, which accounts for 4%, with a smooth surface, concave hila, and invisible lamellae, whose mean size is 8.49 ± 1.62 μm. Meanwhile, compound granule accounts for 9%.

#### 3.2.5 Starch granules from *Lithocarpus* sp.

The starch granules from *Lithocarpus* sp. in our study are divided into three groups according to shape. The first group is the subcircular type, which accounts for 70%, with a smooth surface and invisible lamellae, and with very few granules bearing X-shaped fissures through the hila, whose mean size is 11.88 ± 3.37 μm. The second group is the polygonal type, which accounts for 17%, with a rough surface, concave hila, and invisible lamellae, whose mean size is 11.14 ± 5.49 μm. The last group is the bell-shaped type, which accounts for 12%, with a smooth surface, concave hila, and invisible lamellae, whose mean size is 10.09 ± 3.37 μm. Meanwhile, compound granule accounts for 1%.

#### 3.2.6 Starch granules from Lithocarpus elizabethiae

The starch granules from *Lithocarpus elizabethiae* in our study are divided into two groups according to shape. One is the subcircular type, which accounts for 93%, with a smooth surface, X-shaped or linear-shaped fissures through the hila, and invisible lamellae, whose mean size is 10.21 ± 2.73 μm. The other is the bell-shaped type, which accounts for 6%, with a smooth surface, X-shaped fissures through the hila, and invisible lamellae, whose mean size is 7.22 ± 10.06 μm. Meanwhile, compound granule accounts for 1%.

#### 3.2.7 Starch granules from Lithocarpus longipedicellatus

The starch granules from *Lithocarpus longipedicellatus* in our study are divided into three groups according to shape. The first group is the subcircular type, which accounts for 39%, with a smooth surface and invisible lamellae, whose mean size is 13.19 ± 4.46 μm. The second group is the polygonal type, which accounts for 31%, with a rough surface, concave hila, and invisible lamellae, whose mean size is 17.48 ± 5.71 μm. The last group is the bell-shaped type, which accounts for 30%, with a smooth surface and invisible lamellae, whose mean size is 12.83 ± 5.32 μm. Meanwhile, compound granule accounts for 2%.

#### 3.2.8 Starch granules from *Lithocarpus fenzelianus*

The starch granules from *Lithocarpus fenzelianus* in our study is the subcircular type with a smooth surface and invisible lamellae, whose mean size is 8.35 ± 1.94 μm.

#### 3.2.9 Starch granules from *Lithocarpus balansae*

The starch granules from *Lithocarpus balansae* in our study are divided into three groups according to shape. The first group is the subcircular type, which accounts for 12.2%, with a smooth surface and invisible lamellae, whose mean size is 15.99 ± 3.04 μm. The second group is the polygonal type, which accounts for 41.2%, with a rough surface and visible lamellae, whose mean size is 20.79 ± 3.98 μm. The last group is the oval type, which accounts for 39.7%, with some small hollows on the surface and visible lamellae, whose mean size is 18.16 ± 3.71 μm. Meanwhile, compound granule accounts for 6.9%.

#### 3.2.10 Starch granules from *Lithocarpus skanianus*

The starch granules from *Lithocarpus skanianus* in our study are divided into three groups according to shape. The first group is the subcircular type, which accounts for 7.3%, with some small hollows on the surface and visible lamellae, whose mean size is 14.9 ± 2.81 μm. The second group is the polygonal type, which accounts for 77.6%, with a rough surface and visible lamellae, whose mean size is 19.93 ± 5.2 μm. The last group is the bell-shaped type, which accounts for 12.7%, with a smooth surface and invisible lamellae, whose mean size is 18.66 ± 3.01 μm. Meanwhile, compound granule accounts for 2.4%.

#### 3.2.11 Starch granules from *Lithocarpus longanoides*

The starch granules from *Lithocarpus longanoides* in our study are divided into two groups according to shape. One is the subcircular type, which accounts for 13.5%, with some small hollows on the surface, linear-shaped fissures through the hila, and visible lamellae, whose mean size is 12.63 ± 1.99 μm. The other is the polygonal type, which accounts for 82.5%, with a rough surface, linear-shaped fissures through the hila, and visible lamellae, whose mean size is 15.87 ± 3.34 μm. Meanwhile, compound granule accounts for 4%.

#### 3.2.12 Starch granules from *Lithocarpus xylocarpus*

The starch granules from *Lithocarpus xylocarpus* in our study are divided into two groups according to shape. One is the subcircular type, which accounts for 77.6%, with a smooth surface and invisible lamellae, whose mean size is 12.51 ± 2.95 μm. The other is the oval type, which accounts for 22.4%, with a rough surface and invisible lamellae, whose mean size is 15.76 ± 3.99 μm.

#### 3.2.13 Starch granules from *Lithocarpus longinux*

The starch granules from *Lithocarpus longinux* in our study are divided into two groups according to shape. One is the subcircular type, which accounts for 10.2%, with a smooth surface and invisible lamellae, whose mean size is 7.73 ± 0.88 μm. The other is the bell-shaped type, which accounts for 67%, with a smooth surface and invisible lamellae, whose mean size is 7.69 ± 0.96 μm. Meanwhile, compound granule accounts for 22.8%. The compound granules could be divided into two subtypes: one usually includes two or three small semicircular or fan-shaped single granules, while the other is composed of over four small polygonal single granules.

### 3.3 Starch granules from Castanopsis(*Castanopsis orthacantha*)

The starch granules from *Castanopsis orthacantha* in our study are divided into four groups according to shape. The first group is the subcircular type, which accounts for 17%, with a smooth surface and invisible lamellae, whose mean size is 15.98 ± 3.45 μm. The second group is the polygonal type, which accounts for 65%, with a rough surface and invisible lamellae, whose mean size is 16.67 ± 3.15 μm. The third group is the oval type, which accounts for 8%, with a smooth surface and invisible lamellae, whose mean size is 16.60 ± 2.99 μm. The last group is the bell-shaped type, which accounts for 10%, with a rough surface and invisible lamellae, whose mean size is 12.92 ± 2.16 μm. Meanwhile, compound granule accounts for 2%.

### 3.4 Starch granules from *Castanea* (*Castanea seguinii*)

The starch granules from *Castanea seguinii* in our study are divided into four groups according to shape. The first group is the subcircular type, which accounts for 29%, with a smooth surface and invisible lamellae, whose mean size is 10.34 ± 3.38 μm. The second group is the polygonal type, which accounts for 6%, with a rough surface and invisible lamellae, whose mean size is 14.62 ± 3.25 μm. The third group is the oval type, which accounts for 17%, with a smooth surface and invisible lamellae, whose mean size is 14.65 ± 3.22 μm. The last group is the drop-shaped type, which accounts for 45%, with a smooth surface and invisible lamellae, and with very few granules with concave hila, whose mean size is 12.92 ± 2.16 μm. Meanwhile, compound granule accounts for 3%.

## 4 Discussion

### 4.1 Discrimination of Fagaceae starch granules at the species level

**Figure 3:**
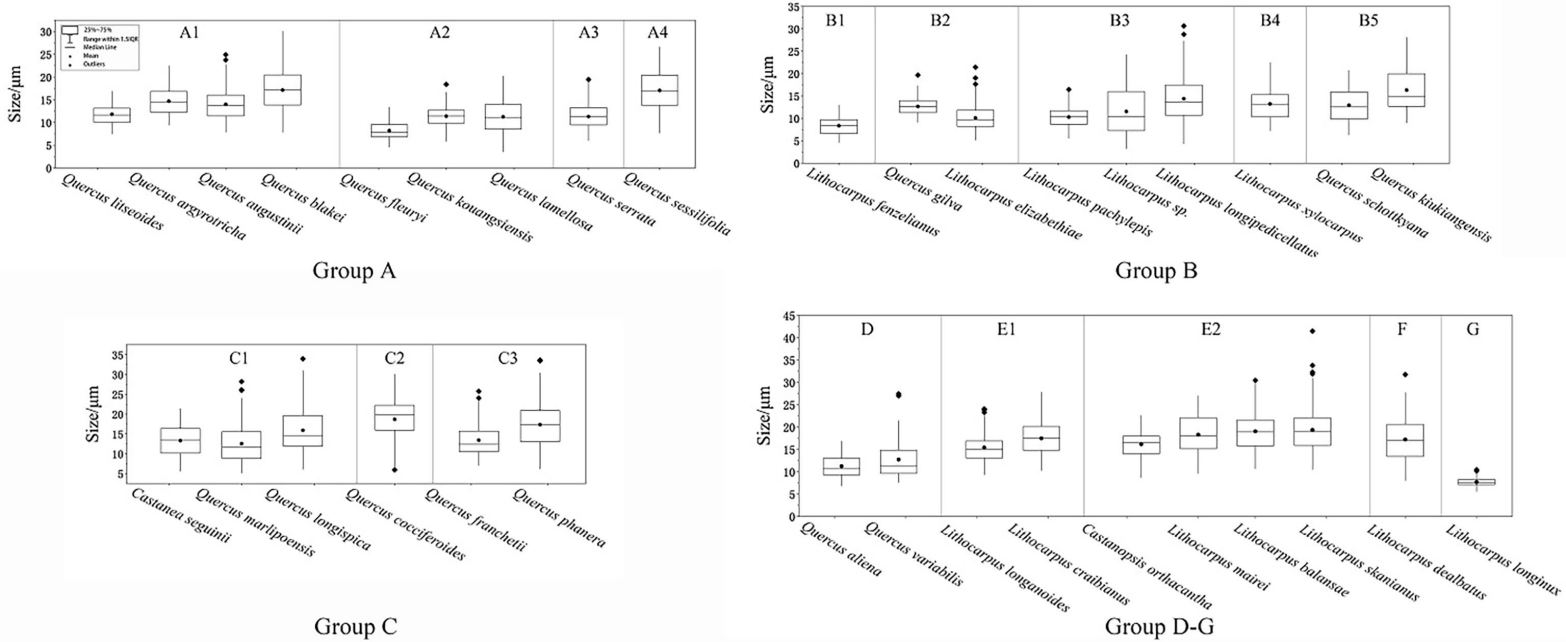
Box plot of the granule sizes of the starches examined.

We statistically measured the granule size as a discriminating feature for starch identification. However, the fact is a lot of granule size overlap makes it challenging to adopt granule size as an independent discriminator. Therefore, size should be used in combination with the following morphological features for reliable starch discrimination: overall shape, fissure types, lamella visibility, hilum position, and surface texture. We divided all specimens into seven groups primarily based on the granule shape, namely, oval, subcircular, drop-shaped, rounded triangle, polygonal, spherical caps, and bell-shaped. Starch granule sizes are given in **Figure 3**.

**Figure 4:**
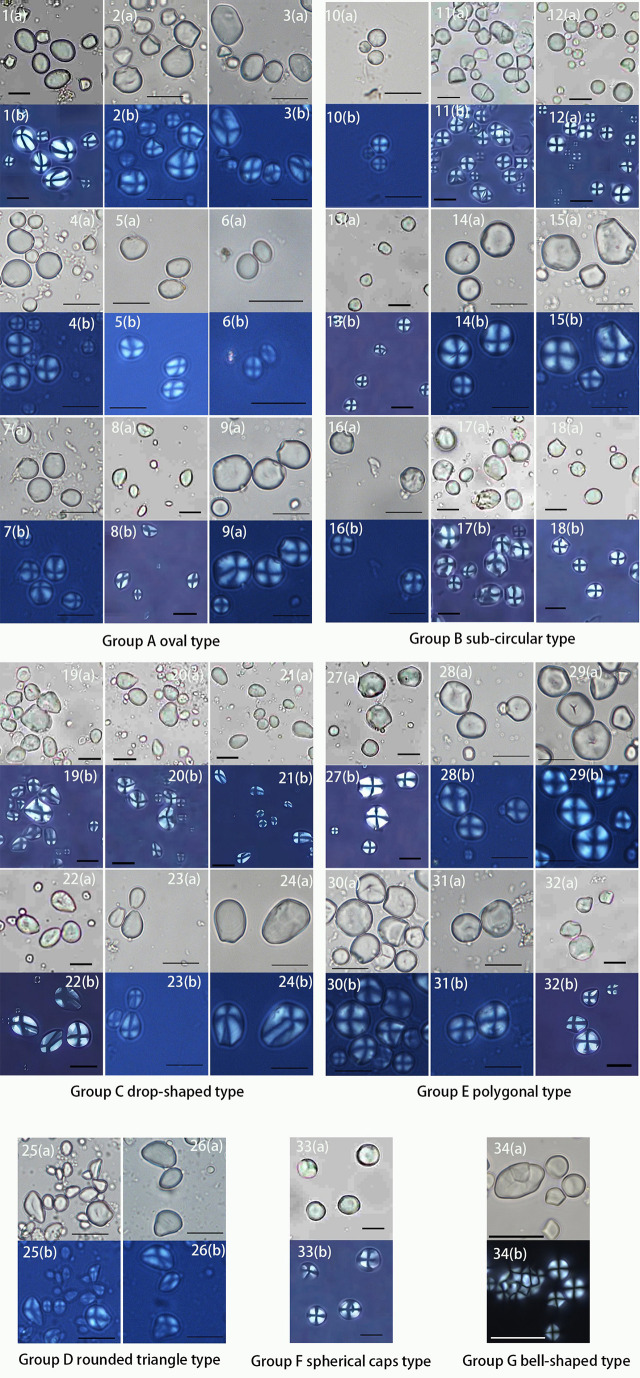
Morphological graphs of 34 species from Fagaceae. Scale bar, 20 μm. (a, brightfield light; b, cross-polarized light). 1, *Quercus blakei;* 2, *Quercus argyrotricha*; 3, *Quercus augustinii*; 4, *Quercus litseoides*; 5, *Quercus kouangsiensis*; 6, *Quercus fleuryi*; 7, *Quercus lamellosa*; 8, *Quercus serrata*; 9, *Quercus sessilifolia*; 10, *Lithocarpus fenzelianus*; 11, *Quercus gilva*; 12, *Lithocarpus elizabethiae*; 13, *Lithocarpus pachylepis*; 14, *Lithocarpus* sp.; 15, *Lithocarpus longipedicellatus*; 16, *Lithocarpus xylocarpus*; 17, *Quercus kiukiangensis*; 18, *Quercus schottkyana*; 19, *Quercus longispica*; 20, *Castanea seguinii*; 21, *Quercus marlipoensis*; 22, *Quercus cocciferoides*; 23, *Quercus franchetii*; 24, *Quercus phanera*; 25, *Quercus aliena*; 26, *Quercus variabilis*; 27, *Lithocarpus craibianus*; 28, *Lithocarpus longanoides*; 29, *Lithocarpus mairei*; 30, *Castanopsis orthacantha*; 31, *Lithocarpus balansae*; 32, *Lithocarpus skanianus*; 33, *Lithocarpus dealbatus*; 34, *Lithocarpus longinux*.

**Figure 5:**
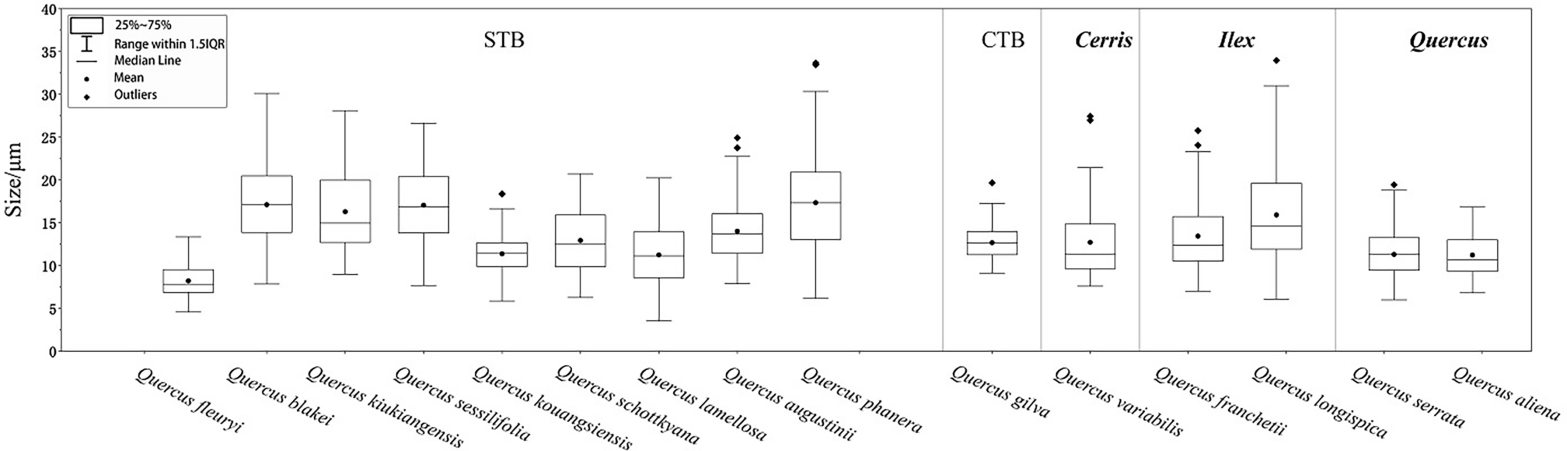
Box plot of the granule sizes of plant materials used for morphological analysis based on phylogeny in this study.

**Figure 6:**
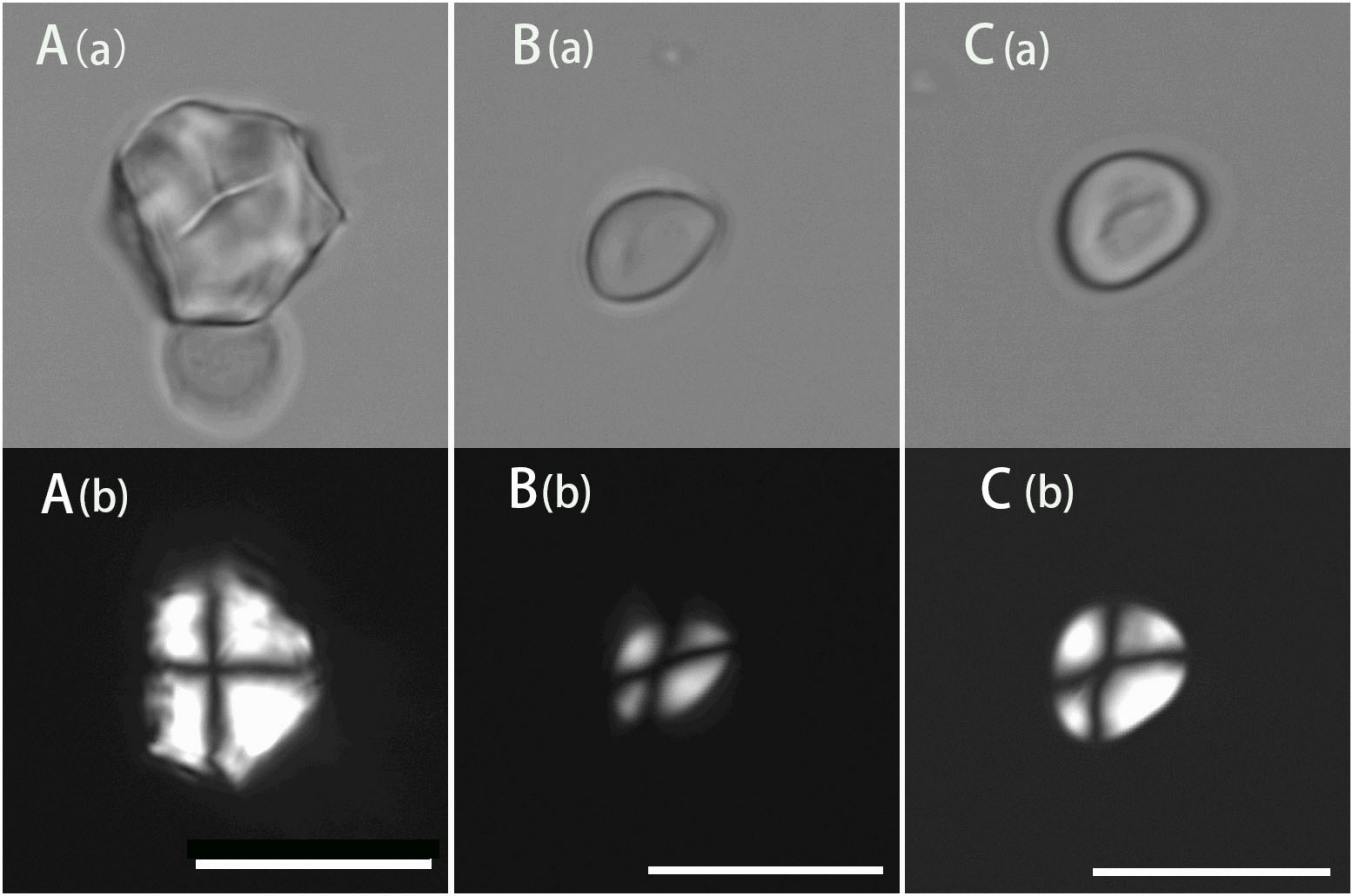
Morphological graphs of starch granules found in the Xiaodong site. Scale bar, 20 μm (a, brightfield light; b, cross-polarized light). **(A)**, ancient starch consistent with Lithocarpus mairei; **(B)**, ancient starch consistent with Quercus serrata; **(C)**, ancient starch consistent with Quercus variabilis and Quercus aliena.

Group A is mainly composed of ovals with an eccentric hilum and invisible lamellae. *Quercus fleuryi* has the smallest starch length in this group at mean length (8.21 ± 1.75 μm), while *Quercus blakei* and *Quercus sessilifolia* are the largest, 17.11 ± 4.43 μm and 17.06 ± 4.46 μm, respectively. *Quercus argyrotricha* and *Quercus augustinii* have similar granule size distributions, as do *Quercus litseoides*, *Quercus kouangsiensis*, and *Quercus serrata.* Group A could be divided into four subtypes based on the type combination. The A1 subtype group contains oval and bell-shaped types; starch granules of *Quercus blakei* (**Figure 4-1**) have a smooth surface, while those of *Quercus argyrotricha* (**Figure 4-2**), *Quercus augustinii* (**Figure 4-3**), and *Quercus litseoides* (**Figure 4-4**) have a rough surface. The A2 subtype group is a combination of oval and subcircular types; *Quercus kouangsiensis* (**Figure 4-5**) contains 12.7% compound granules with some small hollows on the surface. *Quercus fleuryi* (**Figure 4-6**) only contains single granules with a smooth surface. *Quercus lamellosa* (**Figure 4-7**) contains several polygonal types with X-shaped fissures. The A3 subtype group contains *Quercus serrata* (**Figure 4-8**), in which a small part of the rounded triangle type with linear-shaped fissures is included. The A4 subtype group contains *Quercus sessilifolia* (**Figure 4-9**), which only has an oval type with some small hollow surfaces.

The subcircular type dominates in Group B, which has a visible centric hilum and invisible lamella. Group B could be divided into five subtypes based on the type combination. The B1 subtype group contains *Lithocarpus fenzelianus* (**Figure 4-10**), which has the smallest size in Group 2 (8.35 ± 1.94 μm), subcircular type only. The B2 subtype group is a combination of subcircular and bell-shaped types. *Quercus gilva* (**Figure 4-11**) contains 37% compound granules, and some of the single granules have linear fissures, while *Lithocarpus elizabethiae* (**Figure 4-12**) contains almost single granules, some of which have linear fissures or X-shaped fissures. The B3 subtype group is a combination of subcircular, polygonal with wavy edges or convex surfaces, and bell-shaped types. *Lithocarpus pachylepis* (**Figure 4-13**) contains 9% compound granules, and some of the single granules have linear-shaped or X-shaped fissures, while *Lithocarpus* sp. (**Figure 4-14**) has a concave hilum. The statistical results revealed that three types in *Lithocarpus longipedicellatus* (**Figure 4-15**) have an almost equal percentage. The B4 subtype group contains *Lithocarpus xylocarpus* (**Figure 4-16**) with an oval type that has a rough surface. The B5 subtype group contains *Quercus kiukiangensis* (**Figure 4-17**) and *Quercus schottkyana* (**Figure 4-18**), which include oval (concave toward the distal end) and bell-shaped types. The size of *Quercus kiukiangensis* (16.3 ± 4.67 μm) is larger than that of *Quercus schottkyana* (12.93 ± 3.87 μm).

Group C is mainly composed of drop-shaped types with eccentric hila and invisible lamellae. Group C could be divided into three subtypes based on the type combination. The C1 subtype group contains oval, subcircular, and polygonal with wavy edges or convex surfaces. *Quercus longispica* (**Figure 4-19**) and *Castanea seguinii* (**Figure 4-20**) have some compound granules, while they are not observed in *Quercus marlipoensis* (**Figure 4-21**). In addition, the bell-shaped type and oval type, few of which have linear-shaped, V-shaped, and X-shaped fissures, are included in *Quercus longispica*. The C2 subtype group contains *Quercus cocciferoides* (**Figure 4-22**), which includes a considerable number of subcircular types. Some of the granules have linear fissures. The C3 subtype group includes *Quercus franchetii* (**Figure 4-23**) and *Quercus phanera* (**Figure 4-24**), drop-shaped type only. However, *Quercus phanera* (17.35 ± 5.96 μm) is larger than *Quercus franchetii* (13.44 ± 4 μm).

Group D includes *Quercus aliena* (**Figure 4-25**) and *Quercus variabilis* (**Figure 4-26**), in which the rounded triangle type is dominant, with faint polarizing crosses, linear fissures, and invisible lamellae. *Quercus aliena* also contains a few oval types, while *Quercus variabilis* contains several drop-shaped types.

Group E, which is polygonal with wavy edges or convex surfaces, has a mean size larger than 15 μm. Group E could be divided into two subtypes based on the type combination. The E1 subtype group is a combination of polygonal and subcircular types. *Lithocarpus craibianus* (**Figure 4-27**) only contains single granules, while *Lithocarpus longanoides* (**Figure 4-28**) contains few compound granules, and the single one has linear-shaped fissures. The E2 subtype group included more various, polygonal, oval, subcircular, and bell-shaped types. *Lithocarpus mairei* (**Figure 4-29**) and *Castanopsis orthacantha* (**Figure 4-30**) have invisible lamellae, while the former has a concave hilum and linear-shaped or X-shaped fissures. The starch granules of *Lithocarpus balansae* (**Figure 4-31**) and *Lithocarpus skanianus* (**Figure 4-32**) are most likely to have visible lamellae and small hollows on the surface, while the largest size of *Lithocarpus skanianus* could reach 41.47 μm and that of *Lithocarpus balansae* can only reach 30.46 μm.

Group F only contains *Lithocarpus dealbatus* (**Figure 4-33**), which is a combination of spherical caps and polygonal, oval, and subcircular types, and additionally includes 10.6% compound granules. Some starch granules have a concave hilum.

Group G only contains *Lithocarpus longinux* (**Figure 4-34**), which is composed of bell-shaped and subcircular types and contains 22.8% compound granules. The compound granules could be divided into two subtypes as follows: G1 and G2. The former usually includes two or three small semicircular or fan-shaped single granules, while the latter is composed of over four small polygonal single granules. *Lithocarpus longinux* was the smallest among all the specimens (7.7 ± 0.95 μm).

### 4.2 Morphological discrimination on a phylogenetic basis

When compound granules are milled, they can break up into the separate subgranules. Meanwhile, we noted that the species that produced the bell-shaped type almost produced compound granules, and the compound granules are mainly composed of two single bell-shaped granules, which means that the bell-shaped type may originate from the segregative compound granules. There are many folds on the surface of almost all polygonal starch granules. Transverse fissures are present in most *Quercus kiukiangensis, Quercus gilva, Quercus variabilis, Quercus serrata, Quercus aliena, Quercus cocciferoides, Quercus longispica, Lithocarpus mairei, Lithocarpus pachylepis, Lithocarpus elizabethiae*, and *Lithocarpus longanoides* starch granules. Most starch granules of *Lithocarpus longanoides, Lithocarpus skanianus*, and *Lithocarpus balansae* featured lamellae.

Combining the previous work of phylogenetic relationships (Deng et al., 2018; Hipp et al., 2020) with the species tested in this paper, the phylogenetic relationships are given in **Table 2**, and starch granule sizes are given in **Figure 5**.

**Table 2:** Plant materials used for morphological analysis based on phylogeny in this study.

No.	Taxa	Infragenious groups/sections	TB	Length range (μm)	Mean length (μm)
1	*Quercus fleuryi*	*Cyclobalanopsis*	STB	4.57–13.37	8.21 ± 1.75
2	*Quercus blakei*	*Cyclobalanopsis*	STB	7.85–30.1	17.11 ± 4.43
3	*Quercus kiukiangensis*	*Cyclobalanopsis*	STB	8.98–28.1	16.3 ± 4.67
4	*Quercus sessilifolia*	*Cyclobalanopsis*	STB	7.67–26.63	17.26 ± 4.46
5	*Quercus kouangsiensis*	*Cyclobalanopsis*	STB	5.86–18.37	11.38 ± 2.21
6	*Quercus schottkyana*	*Cyclobalanopsis*	STB	6.28–20.73	12.93 ± 3.87
7	*Quercus lamellosa*	*Cyclobalanopsis*	STB	3.53–20.24	11.25 ± 3.72
8	*Quercus augustinii*	*Cyclobalanopsis*	STB	7.87–24.91	14.02 ± 3.52
9	*Quercus phanera*	*Cyclobalanopsis*	STB	6.17–33.67	17.35 ± 5.96
10	*Quercus gilva*	*Cyclobalanopsis*	CTB	9.12–19.68	12.67 ± 1.8
11	*Quercus variabilis*	*Cerris*	West Eurasian Cerris	7.59–27.44	12.71 ± 4.12
12	*Quercus franchetii*	*Ilex*	East Asian Ilex	7.01–25.76	13.44 ± 4.0
13	*Quercus longispica*	*Ilex*	Himalayan subalpine	6.08–33.97	15.92 ± 5.76
14	*Quercus serrata*	*Quercus*	Roburoids	5.99–19.44	11.31 ± 2.65
15	*Quercus aliena*	*Quercus*	Roburoids	6.81–16.83	11.24 ± 2.34

The leaf trichome base (TB) characteristics were obtained from Deng et al. (2014) and Hipp et al. (2020): single-celled trichome base (STB); compound trichome base (CTB). Elev.: elevation.

Starch granule morphology is largely dependent on the genetic composition of the plant, but size and shape can be modified by both the internal and external environments of the plant (Nikuni, 1978; Oliveira et al., 1994; Haase and Plate, 1996). Regardless of environmental factors, the shape and size of the starch granule are often characteristic of the plant taxon. We noticed that all samples in the STB lineage in section *Cyclobalnaopsis* produced oval-type starch granules, which was not observed in the others. Both sections *Ilex* and *Cerris* contain drop-shaped types, which is reasonable because these two sections are close in their phylogenetic relationships. It is a combination of rounded triangle and oval types in two samples of section *Quercus.*

### 4.3 Starch grain analysis of residues from Paleolithic stone tools

The modern starch presentation and comparison of nuts was applied in the identification of starch granules extracted from stone tools from the 20 to 10 ka cultural layer of Xiaodong Rockshelter, which is located in Southwestern Yunnan and the earliest Hoabinhian site discovered so far (Ji et al., 2016). A starch granule extracted from a chopper, which is polygonal with a rough surface and linear-shaped fissures with a size of 18.18 μm, is consistent with *Lithocarpus mairei* in both size and morphology (**Figure 6A**). Another starch granule extracted from a sumatralith, which is rounded triangle with a smooth surface and an X-shaped polarizing cross with a size of 10.7 μm, is consistent with *Quercus serrata* in both size and morphology (**Figure 6B**). Moreover, one starch granule extracted from another sumatralith is consistent with *Quercus variabilis* and *Quercus aliena* in both size and morphology, which is rounded triangle with a smooth surface and linear-shaped fissures with a size of 12.99 μm (**Figure 6C**). Based on the starch granule analysis, it can be inferred that nuts from *Quercus* and *Lithocarpus* were gathered and exploited by ancient people in this region. More starch granules extracted from stone tools in this site show the morphological characteristics of Fagaceae species.

## 5 Conclusion

Although our statistical analysis of granule size overlaps among some species, it still evaluates distribution differences, and combining it with morphological features can help species discrimination. Morphological analysis on a phylogenetic basis shows the discrimination among different groups of phylogeny of *Quercus* section. Different groups tend to produce the same shape starch granules. An application case in archaeology shows that some starch granules from Paleolithic stone tools can be recognized to species level, suggesting that the identification of starches from Fagaceae in our study sample from South China is reliable. Therefore, we confirm the potential for starch granule analysis in archaeology research, which helps to improve the accuracy of the identification of ancient starch and deepen the understanding of plant utilization of ancient humans.

## Data availability statement

The original contributions presented in the study are included in the article/**Supplementary Material**. Further inquiries can be directed to the corresponding author.

## Author contributions

Conceived and designed the experiments: QY, HZ, and XJ. Performed the experiments: TY, NC and WY. Analyzed the data: TY, NC and MD. Wrote the paper: TY, QY and NC. Collected the samples in fieldwork and species identification: MD and KY. All authors contributed to the article and approved the submitted version.

## Funding

This work was jointly supported by the National Natural Science Foundation of China (41991323, U1902208, and 41672344), the Strategic Priority Research Program of Chinese Academy of Sciences (XDB26020301), the Second Tibetan Plateau Scientific Expedition and Research (STEP) (2019QZKK0704), and Yunnan Leading Talent Project (202005AB160008).

## Conflict of interest

The authors declare that the research was conducted in the absence of any commercial or financial relationships that could be construed as a potential conflict of interest.

## Publisher’s note

All claims expressed in this article are solely those of the authors and do not necessarily represent those of their affiliated organizations, or those of the publisher, the editors and the reviewers. Any product that may be evaluated in this article, or claim that may be made by its manufacturer, is not guaranteed or endorsed by the publisher.

## Supplementary material

Supplementary Table 1 Plant materials used in this study and their collection information.

Supplementary Table 2 Dichotomous key for starch granule of Fagaceae.
