# Supplementary material for: Morphological characteristics of seed starch granules of Fagaceae in South China and their implication in paleodiet
Source: Front Plant Sci. 2022 Nov 14;13:977152. doi: 10.3389/fpls.2022.977152 (PMC9702991; doi:10.3389/fpls.2022.977152)
Supplement: Supplementary Table 1 — Plant materials used in this study and their collection information. [file Table_1.doc]

**Table S1 Plant materials used in this study and their collection information.**

| Family | Genus | Species | Sample source | Sample number | Date | Elev. (m) | GPS |
| --- | --- | --- | --- | --- | --- | --- | --- |
| Fagaceae | *Quercus* | *Quercus blakei* | Limu, Wuzhou, Guangxi, China | DM23708 | 20210520 | 184 | 22°28′28.9″N  111°4′19.5″E |
| Fagaceae | *Quercus* | *Quercus gilva* | Ji’an, Jiangxi, China | DM23331 | 20201109 | 800 | 27°23′9.7″N  114°36′12.3″E |
| Fagaceae | *Quercus* | *Quercus kiukiangensis* | De’ergong, Linzhi, Tibe, China | DM23562 | 20201209 | 1969 | 29°10′48.2″N  95°8′38.4″E |
| Fagaceae | *Quercus* | *Quercus litseoides* | Wu-tong-shan reserve, Guangdong, China | DM24834 | 20211101 | 911 | 22°34′53.3″N  114°12′58.6″ |
| Fagaceae | *Quercus* | *Quercus augustinii* | Yuping, Honghe, Yunnan, China | DM24604 | 20211020 | 2036 | 22°54′47.5″N  103°41′49.8″E |
| Fagaceae | *Quercus* | *Quercus lamellosa* | Gedang village, Linzhi, Tibet , China | DM23616 | 20201223 | 1669 | 29°30′31.7″N  95°29′9.6″E |
| Fagaceae | *Quercus* | *Quercus fleuryi* | Dongsi foresty park, Hainan, China | DM25206 | 20211106 | 1080 | 19°5′23.1″N  109°11′30.0″E |
| Fagaceae | *Quercus* | *Quercus phanera* | Limu, Wuzhou, Guangxi, China | DM23710 | 20210520 | 184 | 22°28′28.9″N  111°4′19.5″E |
| Fagaceae | *Quercus* | *Quercus sessilifolia* | Ji’an, Jiangxi, China | DM23329 | 20201109 | 800 | 27°23′9.7″N  114°36′12.3″E |
| Fagaceae | *Quercus* | *Quercus argyrotricha* | Guishan national nature reserve, Shilin, Kunming, Yunnan Province | DM24418 | 20211004 | 2239 | 24°38′29.9″N  103°35′45.6″E |
| Fagaceae | *Quercus* | *Quercus kouangsiensis* | Anping, Cenxi, Guangxi, China | DM25125 | 20211012 |  |  |
| Fagaceae | *Quercus* | *Quercus rex* | Puwen, Yunnan, China | DM25163 |  | 923.13 | 22°26′14.1″N  101°1′26.9″E |
| Fagaceae | *Quercus* | *Quercus patelliformis* | Ba-wang-lin national nature reserve, Changjiang, Hainan, China | DM24967 | 20211104 | 603 | 19°4′50.7″N  109°7′22.1″E |
| Fagaceae | *Quercus* | *Quercus schottkyana* | Kunming Institute of Botany, Kunming, Yunnan, China | DM24225 | 20210909 | 1970 | 25°50′12.8″N  102°26′35.1″E |
| Fagaceae | *Quercus* | *Quercus franchetii* | Wshan village, Honghe, Yunnan, China | DM22973 | 20201025 | 1881 | 24°10′25.2″N  103°14′4.6″E |
| Fagaceae | *Quercus* | *Quercus variabilis* | Kunming Institute of Botany, Kunming, Yunnan, China | DM24226 | 20210909 | 1970 | 25°50′12.8″N  102°26′35.1″E |
| Fagaceae | *Quercus* | *Quercus cocciferoides* | Miyang, Honghe, Yunnan, China | DM22459 | 20201024 | 1321 | 24°25′1.8″N  103°26′3.5″E |
| Fagaceae | *Quercus* | *Quercus serrata* | Kunming Institute of Botany, Kunming, Yunnan, China | DM24227 | 20210909 | 1970 | 25°50′12.8″N  102°26′35.1″E |
| Fagaceae | *Quercus* | *Quercus marlipoensis* | Malipo, Wenshan, Yunnan, China | DM24292 | 20210925 | 1530 | 23°24′30.9″N  104°42′32.4″E |
| Fagaceae | *Quercus* | *Quercus aliena* | Kunming Institute of Botany, Kunming, Yunnan, China | DM24224 | 20210909 | 1970 | 25°50′12.8″N  102°26′35.1″E |
| Fagaceae | *Quercus* | *Quercus longispica* | Dashao village Kunming, Yunnan, China | DM23749 | 20210612 | 2310 | 25°7′24.5″N  102°26′36.6″E |
| Fagaceae | *Lithocarpus* | *Lithocarpus balansae* | Malipo, Wenshan, Yunnan, China | DM24482 | 20211003 |  |  |
| Fagaceae | *Lithocarpus* | *Lithocarpus craibianus* | Wumeng, Luquan, Kunming, Yunnan, China | DM24276 | 20210923 | 2460 | 26°2′45.6″N  102°48′54.5″E |
| Fagaceae | *Lithocarpus* | *Lithocarpus dealbatus* | Liangshan, Chuxiong, Yunnan, China | DM23137 | 20201031 | 2557 | 25°26′4.0″N  101°34′25.7″E |
| Fagaceae | *Lithocarpus* | *Lithocarpus mairei* | Pingdian, Yuxi, Yunnan, China | DM24048 |  |  |  |
| Fagaceae | *Lithocarpus* | *Lithocarpus c.f. annamitorus* | Malipo, Wenshan, Yunnan, China | DM23059 | 20201014 | 1000 | 23°7′6.9″N  104°49′0.9″E |
| Fagaceae | *Lithocarpus* | *Lithocarpus longipedicellatus* | Ba-wang-lin national nature reserve, Changjiang, Hainan, China | DM25277 | 20211107 | 0 | 19°7′27.6″N  109°9′23.1″E |
| Fagaceae | *Lithocarpus* | *Lithocarpus elizabethiae* | Kunming Institute of Botany, Kunming, Yunnan, China |  | 20210909 | 1970 | 25°50′12.8″N  102°26′35.1″E |
| Fagaceae | *Lithocarpus* | *Lithocarpus pachylepis* | Yuping, Honghe, Yunnan, China | DM24515 | 20211018 | 1270 | 22°56′40.8″N  103°41′47.8″E |
| Fagaceae | *Lithocarpus* | *Lithocarpus fenzelianus* | Dongsi foresty park, Hainan, China | DM25220 | 20211107 | 1117 | 19°5′29.3″N  109°11′37.5″E |
| Fagaceae | *Lithocarpus* | *Lithocarpus longanoides* | Bajia, Yangchun, Guangdong, China | DM25123 | 20211111 | 597 | 21°53′34.0″N  111°25′37.9″E |
| Fagaceae | *Lithocarpus* | *Lithocarpus bacgiangensis* | Malipo, Wenshan, Yunnan, China | DM22965 | 20201014 | 1200 | 23°8′18.9″N  104°44′12.0″E |
| Fagaceae | *Lithocarpus* | *Lithocarpus gymnocarpus* | Ma’andi, Honghe, YunnanChina | DM24656 | 20211020 | 1211 | 22°46′35.5″N  103°29′12.1″E |
| Fagaceae | *Lithocarpus* | *Lithocarpus longinux* | Malipo, Wenshan, Yunnan, China | DM24347 | 20210929 | 1057 | 23°7′43.0″N  104°42′1.4″E |
| Fagaceae | *Lithocarpus* | *Lithocarpus skanianus* | Yanling, Zhuzhou, Hunan, China | DM24718 | 20211018 | 930 | 26°14′37.6″N  113°55′49.4″E |
| Fagaceae | *Lithocarpus* | *Lithocarpus xylocarpus* | Yuping, Honghe, Yunnan, China | DM24594 | 20211020 | 2090 | 22°54′28.8″N  103°41′45.8″E |
| Fagaceae | *Lithocarpus* | *Lithocarpus sp.* | Malipo, Wenshan, Yunnan, China | DM24475 | 20211103 |  |  |
| Fagaceae | *Lithocarpus* | *Lithocarpus sp.* | Wuzhishan reserve, Hainan, China | DM24903 | 20211103 | 877 | 18°54′11.7″N  109°41′5.0″E |
| Fagaceae | *Castanea* | *Castanea seguinii* | Kunming Institute of Botany, Kunming, Yunnan, China |  | 20210909 | 1970 | 25°50′12.8″N  102°26′35.1″E |
| Fagaceae | *Castanopsis* | *Castanopsis orthacantha* | Kunming Institute of Botany, Kunming, Yunnan, China |  | 20210909 | 1970 | 25°50′12.8″N  102°26′35.1″E |
