# Supplementary material for: Morphological characteristics of seed starch granules of Fagaceae in South China and their implication in paleodiet
Source: Front Plant Sci. 2022 Nov 14;13:977152. doi: 10.3389/fpls.2022.977152 (PMC9702991; doi:10.3389/fpls.2022.977152)
Supplement: Supplementary Table 2 — Dichotomous key for starch granule of Fagaceae. [file Table_2.doc]

**Table 2 Plant materials used for morphological analysis based on phylogent in this study**

| No. | Taxa | Infragenious groups/sections | TB | Length range/μm | Mean length/μm |
| --- | --- | --- | --- | --- | --- |
| 1 | *Quercus fleuryi* | *Cyclobalanopsis* | STB | 4.57-13.37 | 8.21±1.75 |
| 2 | *Quercus blakei* | *Cyclobalanopsis* | STB | 7.85-30.1 | 17.11±4.43 |
| 3 | *Quercus kiukiangensis* | *Cyclobalanopsis* | STB | 8.98-28.1 | 16.3±4.67 |
| 4 | *Quercus sessilifolia* | *Cyclobalanopsis* | STB | 7.67-26.63 | 17.26±4.46 |
| 5 | *Quercus kouangsiensis* | *Cyclobalanopsis* | STB | 5.86-18.37 | 11.38±2.21 |
| 6 | *Quercus schottkyana* | *Cyclobalanopsis* | STB | 6.28-20.73 | 12.93±3.87 |
| 7 | *Quercus lamellosa* | *Cyclobalanopsis* | STB | 3.53-20.24 | 11.25±3.72 |
| 8 | *Quercus augustinii* | *Cyclobalanopsis* | STB | 7.87-24.91 | 14.02±3.52 |
| 9 | *Quercus phanera* | *Cyclobalanopsis* | STB | 6.17-33.67 | 17.35±5.96 |
| 10 | *Quercus gilva* | *Cyclobalanopsis* | CTB | 9.12-19.68 | 12.67±1.8 |
| 11 | *Quercus variabilis* | *Cerris* | West Eurasian Cerris | 7.59-27.44 | 12.71±4.12 |
| 12 | *Quercus franchetii* | *Ilex* | East Asian Ilex | 7.01-25.76 | 13.44±4.0 |
| 13 | *Quercus longispica* | *Ilex* | Himalayan subalpine | 6.08-33.97 | 15.92±5.76 |
| 14 | *Quercus serrata* | *Quercus* | Roburoids | 5.99-19.44 | 11.31±2.65 |
| 15 | *Quercus aliena* | *Quercus* | Roburoids | 6.81-16.83 | 11.24±2.34 |

The leaf trichome base (TB) characteristics were obtained from Deng et al. (2014) and Hipp et al. (2020): single-celled trichome base (STB); compound trichome base (CTB). Elev.: elevation.
